# Supplementary material for: miR200-regulated CXCL12β promotes fibroblast heterogeneity and immunosuppression in ovarian cancers
Source: Nat Commun. 2018 Mar 13;9:1056. doi: 10.1038/s41467-018-03348-z (PMC5849633; doi:10.1038/s41467-018-03348-z)
Supplement: Supplementary file 1 — Supplementary Information [file 41467_2018_3348_MOESM1_ESM.pdf]

**SUPPLEMENTARY INFORMATION for**

# miR200-regulated CXCL12β promotes fibroblast heterogeneity and immunosuppression in ovarian cancers

Anne-Marie Givel[1,2,], Yann Kieffer[1,2,], Alix Scholer-Dahirel[1,2],
Philemon Sirven[1,2,3], Melissa Cardon[1,2], Floriane Pelon[1,2], Ilaria Magagna[1,2]
Géraldine Gentric[1,2], Ana Costa[1,2], Claire Bonneau[1,2], Virginie Mieulet[1,2],
Anne Vincent-Salomon[4] and Fatima Mechta-Grigoriou[1,2,*]

Running title: CAF subsets and Immunosuppression in HGSOC

*Corresponding author: fatima.mechta-grigoriou@curie.fr

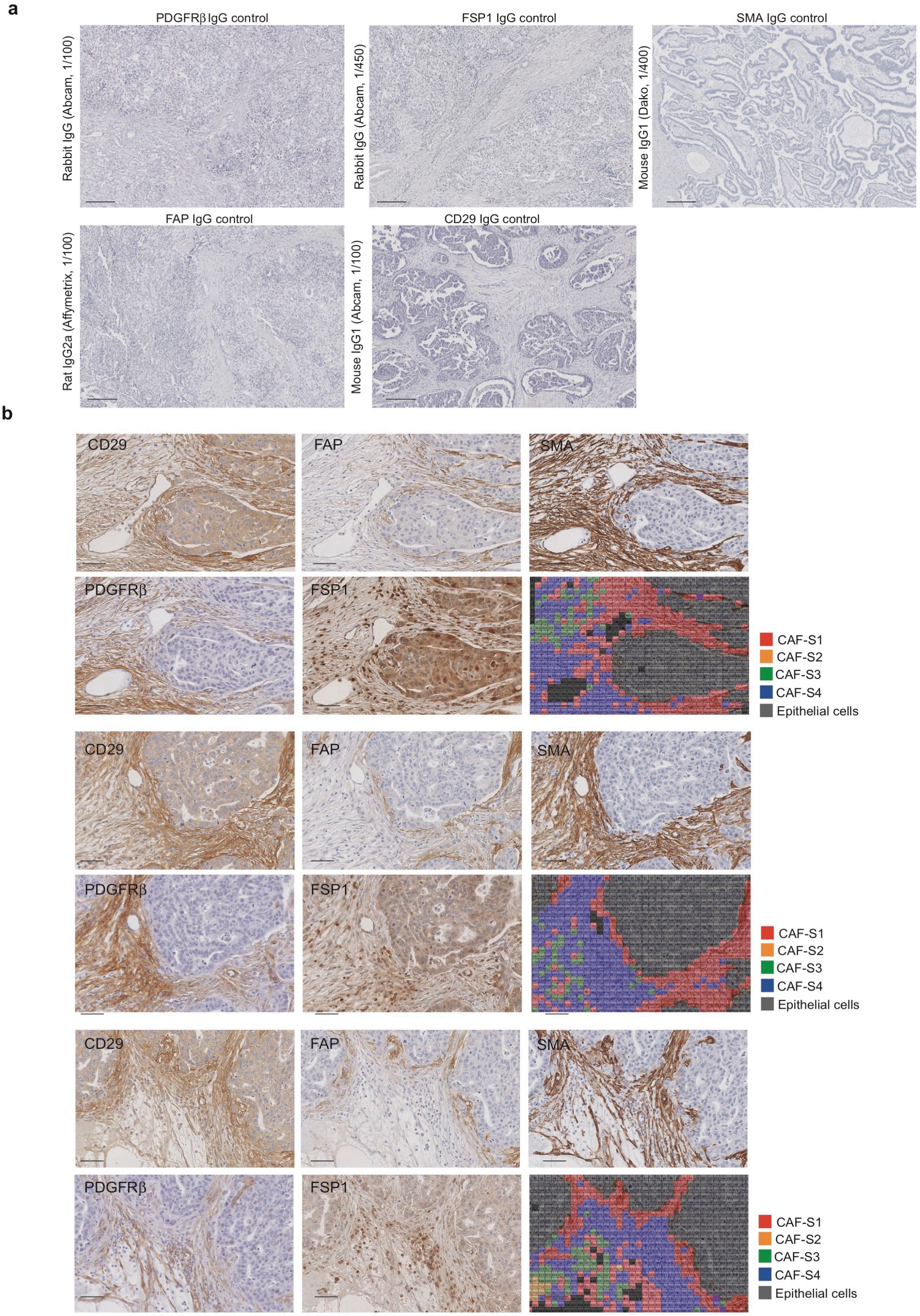


**Supplementary Figure 1** CAF markers specificity and CAF maps from HGSOC sample patients. **a** Representative views of immunostaining performed using IgG control antibodies corresponding to the 5 CAF markers, as indicated, in the exact same conditions as used with

the corresponding specific antibodies. Scale bar = 200 µm. **b** Maps of CAF subsets at cellular level (Bottom Right), with the corresponding views of CD29, FAP, SMA, PDGFRβ and FSP1 immunostaining of HGSOC serial sections, as indicated. For CAF maps, each square of 225 µm$^2$ correspond on average to a single cell and each CAF subset is represented by a color code, as indicated, with CAF-S1 in red, CAF-S2 in orange, CAF-S3 in green, CAF-S4 in blue and epithelial cells in grey. These images were reconstructed by applying mathematical modeling (see Methods) on CAF markers histological scores on serial sections. Scale bar = 200 µm.

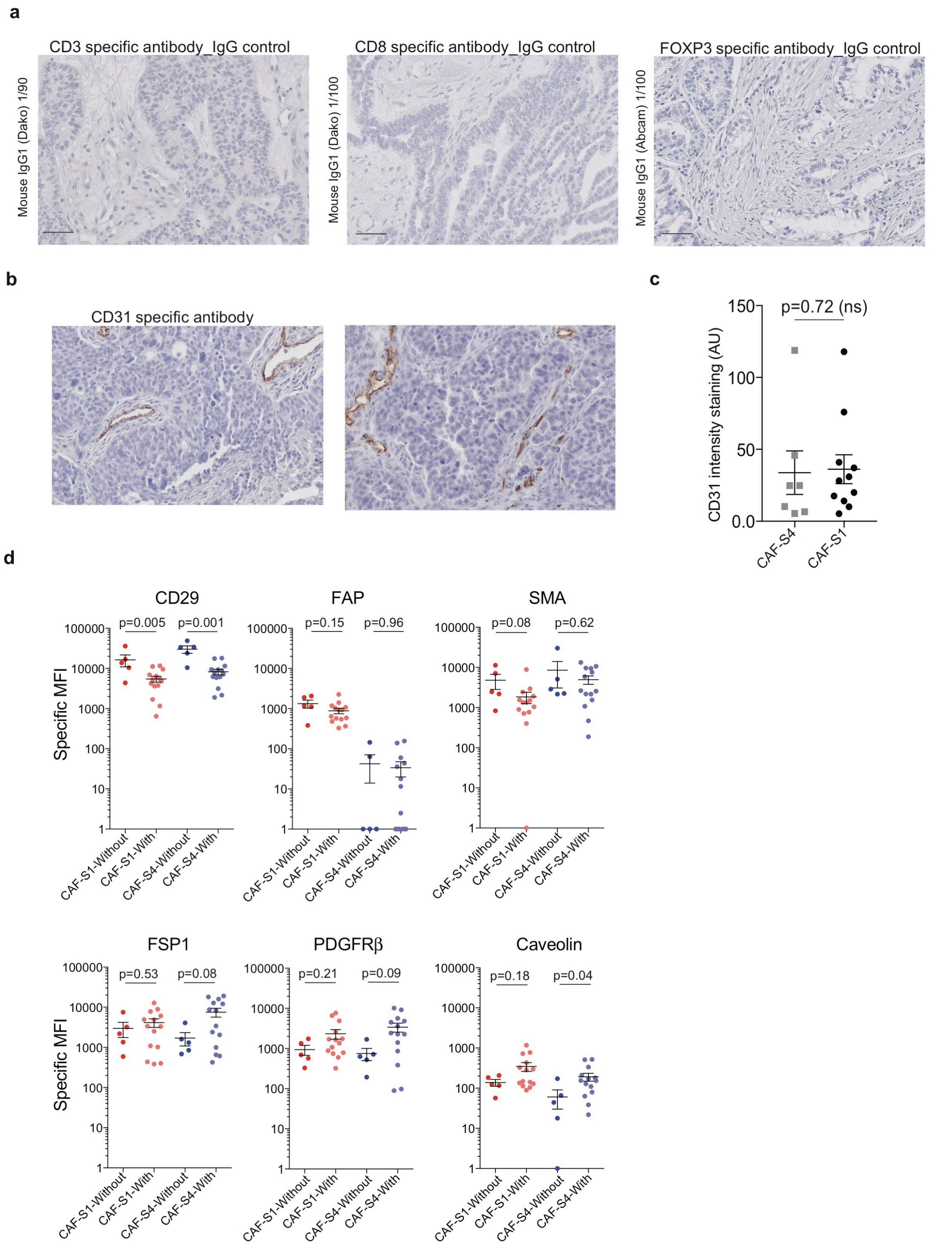


**Supplementary Figure 2** Immune antibodies specificity and vascular content in HGSOC. **a** Representative views of immunostaining (IHC) performed using IgG control antibodies corresponding to the immune cell markers, as indicated, in the exact same conditions as used with the corresponding specific antibodies. Scale bar = 200 µm. **b** Representative views

of CD31+ blood vessels, detected by using CD31+ specific staining, in HGSOC sections. **c** Scatter plot showing the quantification of CD31+ blood vessels in CAF-S1 and CAF-S4 enriched HGSOC samples (*N* = 18). Quantification was performed by densitometry analysis of CD31+ endothelial cells on HGSOC sections using ImageJ software. Data are shown as mean ± SEM. *P* value is from Mann-Whitney test. **d** Scatter plot comparing CAF maker intensities detected in CAF-S1 and CAF-S4 subsets identified by FACS from HGSOC biopsies with or without neoadjuvant chemotherapy, as indicated. Data are expressed as specific MFI (Mean Fluorescent Intensity). Data are from 18 patients and are shown as mean ± SEM. *P* values are from Mann-Whitney test.

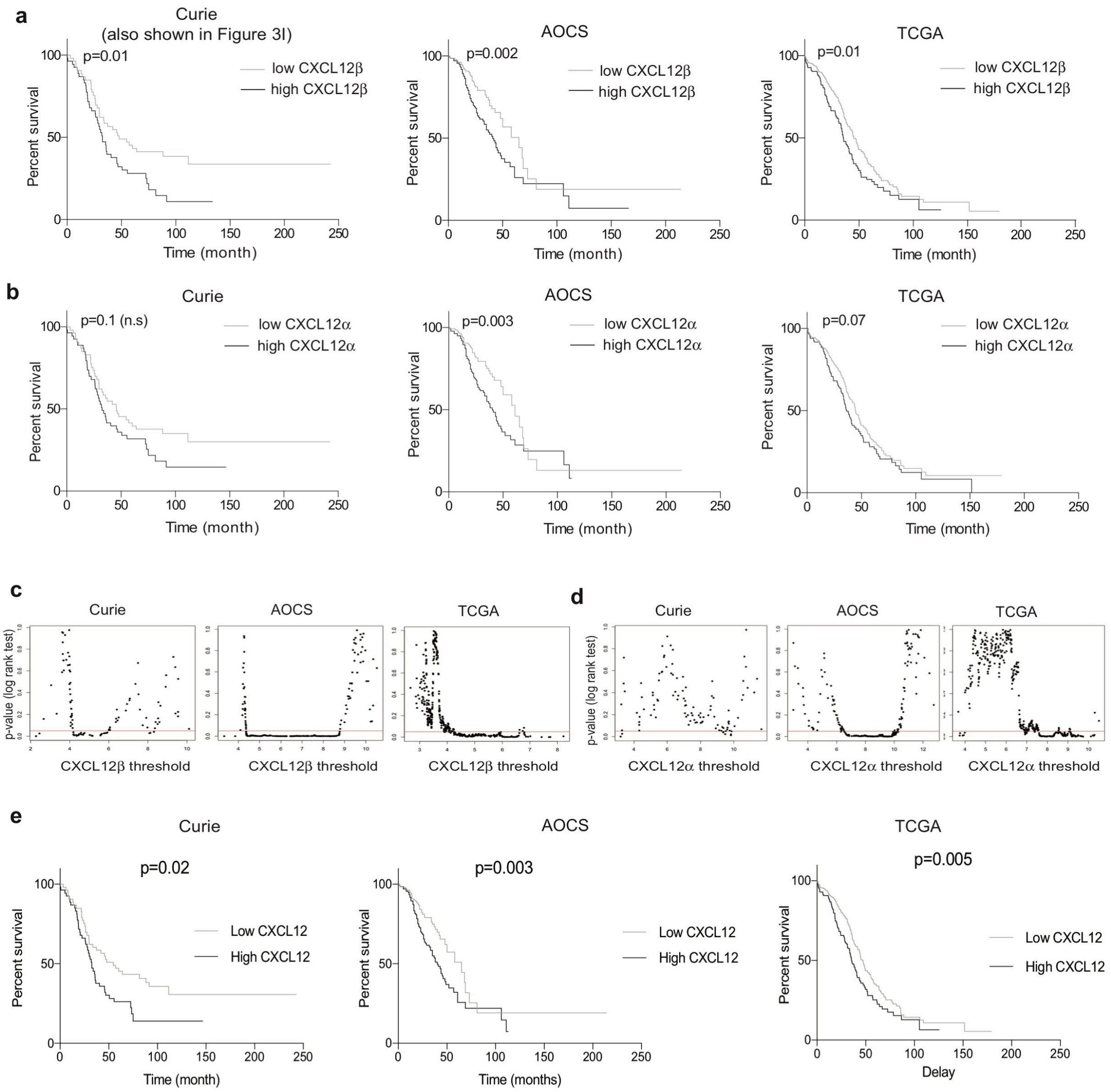


f

$p=10^{-8}$

| Curie | Low CXCL12β | High CXCL12β | Total |
|---|---|---|---|
| Non-mesenchymal | 40 (76%) | 11 (20%) | 51 |
| Mesenchymal | 13 (24%) | 43 (80%) | 56 |
| Total | 53 (100%) | 54 (100%) | 107 |

$p=1.2 \times 10^{-8}$

| AOCS | Low CXCL12β | High CXCL12β | Total |
|---|---|---|---|
| Non-mesenchymal | 111 (86%) | 37 (24%) | 148 |
| Mesenchymal | 18 (14%) | 116 (76%) | 134 |
| Total | 129 (100%) | 153 (100%) | 282 |

$p=2.6 \times 10^{-23}$

| TCGA | Low CXCL12β | High CXCL12β | Total |
|---|---|---|---|
| Non-mesenchymal | 264 (74%) | 52 (29%) | 316 |
| Mesenchymal | 93 (26%) | 127 (71%) | 220 |
| Total | 357 (100%) | 179 (100%) | 536 |

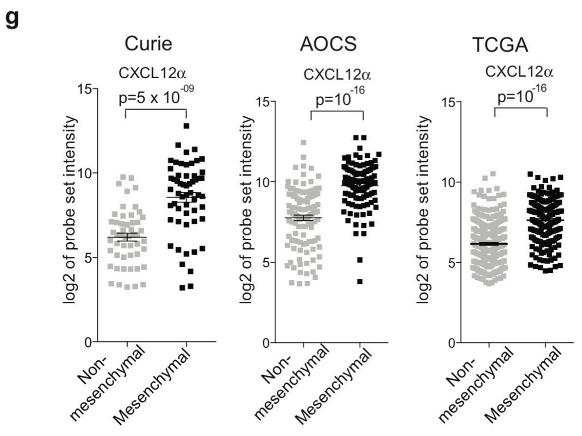


**Supplementary Figure 3** mRNA level of *CXCL12β*, but not *CXCL12α*, is associated with poor survival in 3 independent cohorts of HGSOC.

**a** Kaplan-Meier curves of overall survival according to *CXCL12β* mRNA high and low expression using the optimal threshold determined with iteration tests (c) in Institut Curie cohort (left panel, *N* = 53 for low-CXCL12β expression and *N* = 54 for high-CXCL12β expression); in AOCS cohort (middle panel, *N* = 141 for low-CXCL12β expression and *N* = 141 for high-CXCL12β expression); and in TCGA cohort (right panel, *N* = 305 for low-CXCL12β expression and *N* = 153 for high-CXCL12β expression). *P* values are from Log-Rank test. **b** Kaplan-Meier curves of overall survival according to *CXCL12α* mRNA high and low expression using the optimal threshold determined with iteration tests (d) in Institut Curie cohort (left panel, *N* = 53 for low-CXCL12α expression and *N* = 54 for high-CXCL12α expression); in AOCS cohort (middle panel, *N* = 141 for low-CXCL12α expression and *N* = 141 for high-CXCL12α expression); and in TCGA cohort (right panel, *N* = 305 for low-CXCL12α expression and *N* = 153 for high-CXCL12α expression). *P* values are from Log-Rank test. **c** Scatter plots showing *P* values of log-rank test computed by successive iterations for each threshold value of *CXCL12β* expression in Curie (Left), AOCS (middle) or TCGA (right) cohorts. **d** Scatter plots showing *P* values of log-rank test computed by successive iterations for each threshold value of *CXCL12α* expression in Curie (Left), AOCS (middle) or TCGA (right) cohorts. **e** Kaplan-Meier curves of overall survival according to *CXCL12* mRNA high and low expression in Institut Curie cohort (left panel, *N* = 53 for low-CXCL12 expression and *N* = 54 for high-CXCL12 expression); in AOCS cohort (middle panel, *N* = 141 for low-CXCL12 expression and *N* = 141 for high-CXCL12 expression); and in TCGA cohort (right panel, *N* = 305 for low-CXCL12 expression and *N* = 153 for high-CXCL12 expression). *P* values are from Log-Rank test. **f** Contingency tables showing association between mesenchymal/non-mesenchymal and high/low-CXCL12β subgroups in HGSOC. Data are from Curi (Up), AOCS (Middle) and TCGA (Down) HGSOC patient cohorts. *P* values are from Fisher's Exact test. **g** Box plots showing *CXCL12α* mRNA levels in mesenchymal/non-mesenchymal HGSOC from the Institut Curie, AOCS and TCGA cohorts, as indicated. Expression data are from Affymetrix U133 plus2.0 micro-arrays for Institut Curie and AOCS cohorts and from Affymetrix U133A micro-array (Level 2) for TCGA. 209687_at probeset detects *CXCL12α* mRNA. Values are expressed in log2 of probeset intensity. Data are shown as mean ± SEM. *P* values are from Student's t-test.

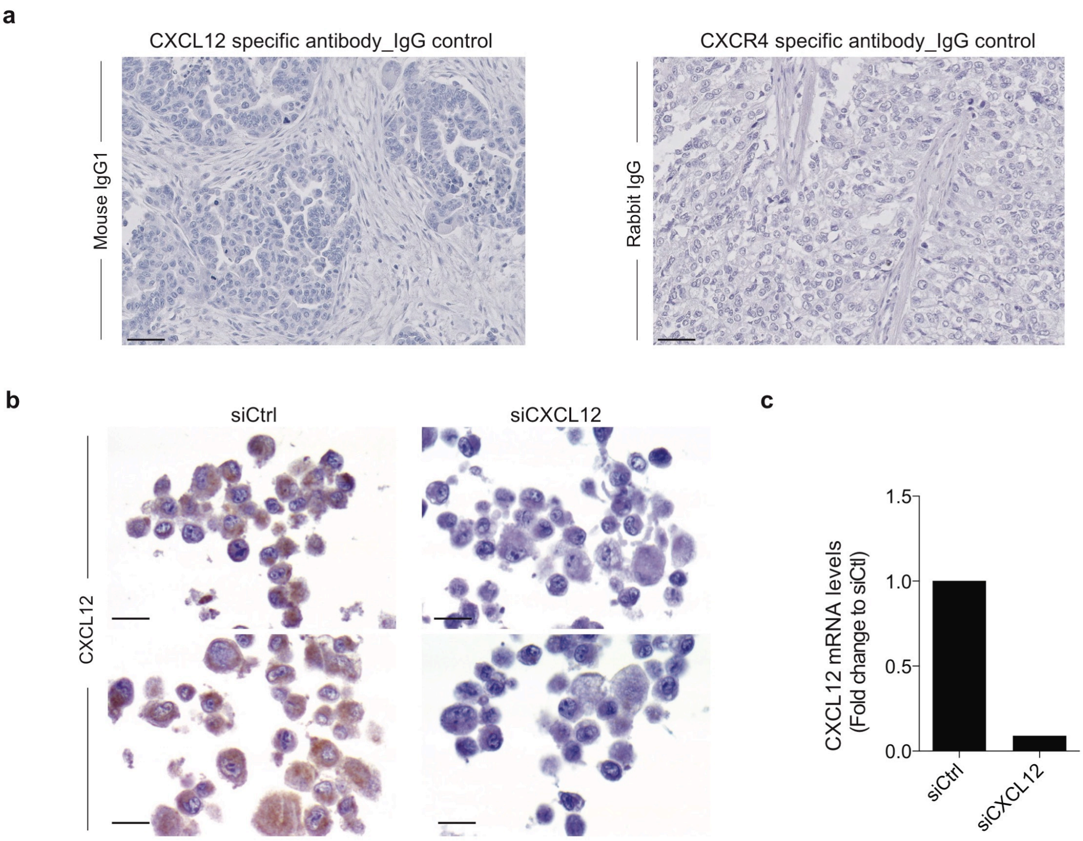


**Supplementary Figure 4** Specificity of CXCL12 and CXCR4 antibodies used for immunohistochemistry analyses of ovarian tumor sections. **a** Representative views of ovarian tumor immunostaining using IgG control antibody corresponding to CXCL12 antibody (left panel) and CXCR4 antibody (right panel). Immunostaining were performed with the exact same conditions as used for staining with CXCL12- or CXCR4-specific antibodies. Scale bar = 50 µm. **b** Representative view of CXCL12 immunostaining on sections from paraffin-embedded fibroblast cells after transfection either with control siRNA (siCtrl, left panels) or with CXCL12-targeting siRNA (siCXCL12, right panel) in order to test the specificity of the antibody used. Scale bar = 20 µm **c** Bar plot showing *CXCL12* mRNA levels assessed by qPCR, in the same cells as in (b) to verify transfection efficiency.

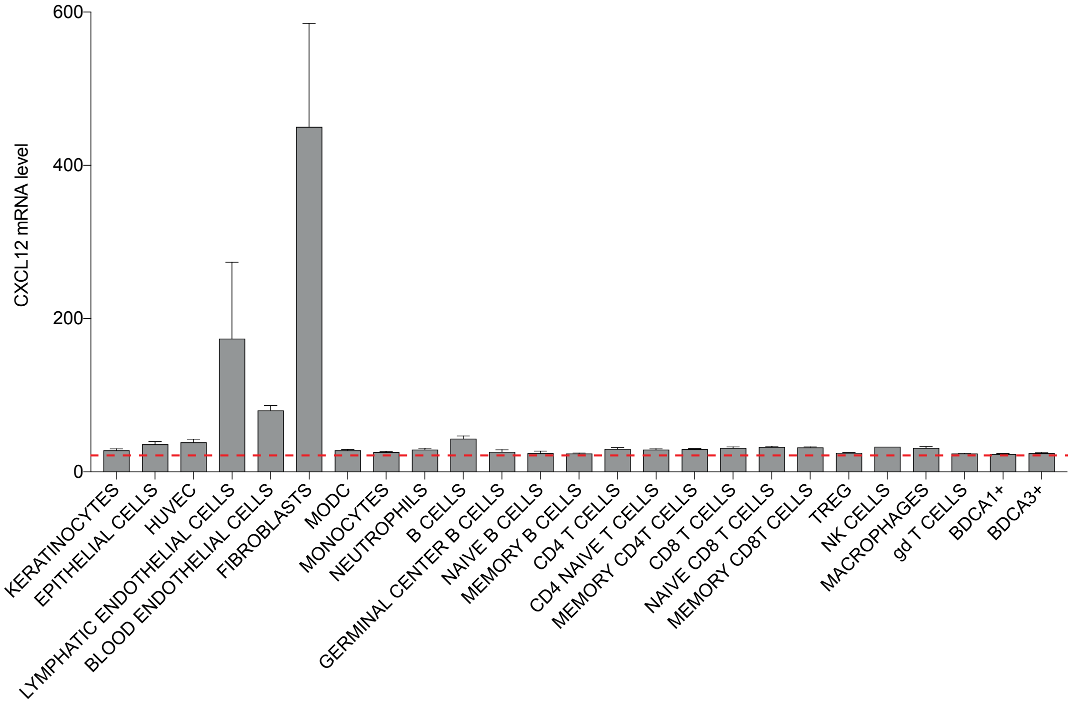


**Supplementary Figure 5** *CXCL12β* expression profile among different cell types. Bar plot showing *CXCL12* mRNA level in various primary cell lines, as indicated. Data have been generated from Affymetrix U133plus2.0 array and are publically available on GEO under accession number GSE49910. *CXCL12* mRNA level is shown as the average of expression of 203666_at and 209687_at probesets, representative of CXCL12β and *CXCL12α,* respectively. The red dashed line indicates limit of detection of Affymetrix U133plus2.0 array.

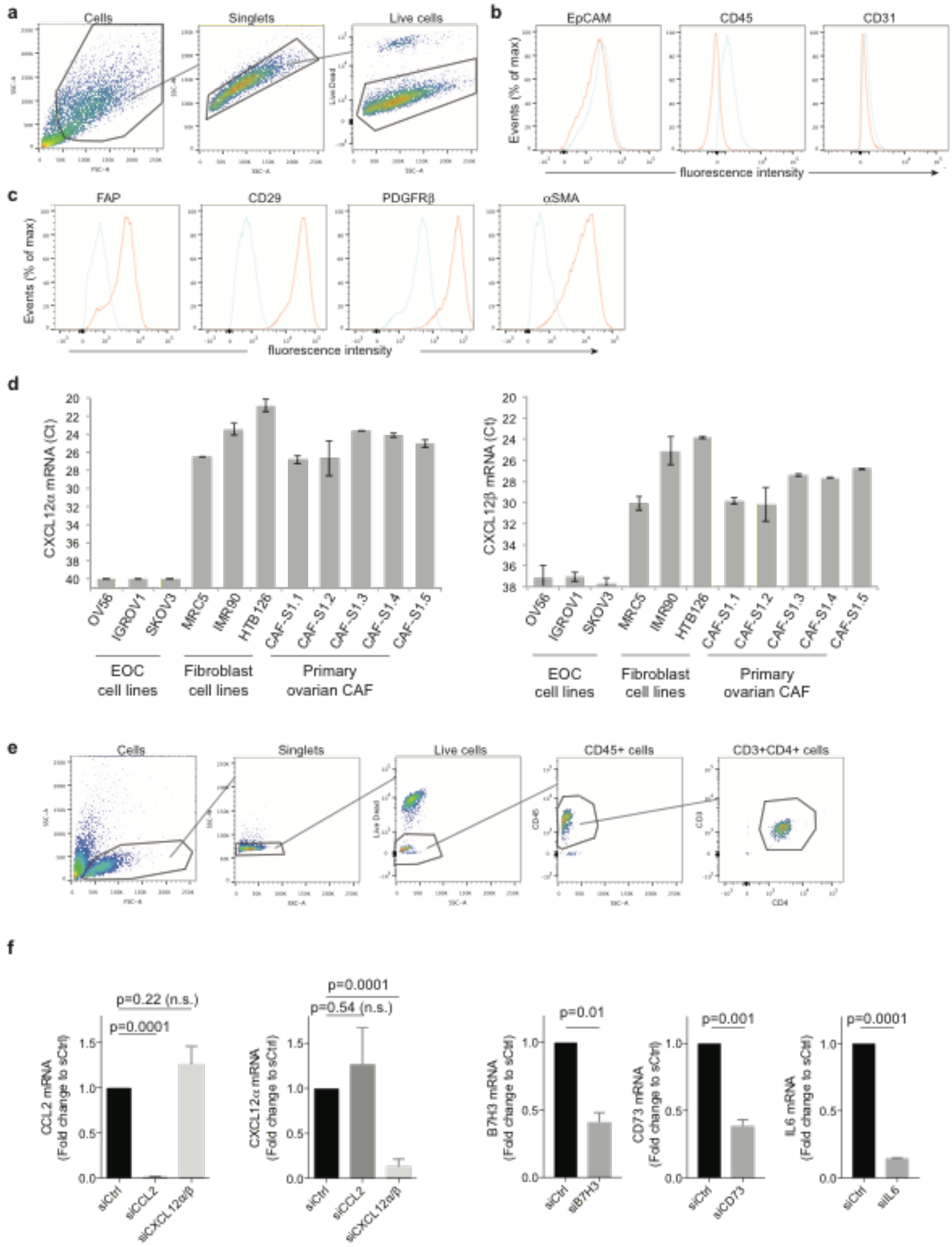


**Supplementary Figure 6** Characterization of human primary CAF-S1 fibroblasts and T lymphocytes. **a** Gating strategy used to characterize primary ovarian CAF. Debris and doublets were excluded based on SSC-A (side scatter), FSC-A (forward scatter) parameters,

and SSC-A, SSC-W parameters respectively. Dead cells were excluded based on their positive staining for Live/Dead marker. **b** Curves showing the fluorescence intensity detected for EPCAM, CD45 and CD31 in order to confirm that primary cells are not epithelial, hematopoietic or endothelial cells, respectively. Specific staining (red) was compared to isotype control (blue). **c** Curves showing the fluorescence intensity detected for FAP, CD29, PDGFRβ and αSMA (red) markers relative to the isotype control (blue). Primary CAF expresses high level of FAP, CD29, PDGFRβ and αSMA, which identify it as CAF-S1 subtype. **d** Bar plot showing *CXCL12α* (Left) and *CXCL12β* (Right) mRNA levels in epithelial ovarian cancer cell lines, immortalized fibroblasts cell lines and primary CAF isolated from fresh HGSOC. Values are expressed in $2^{-\Delta Ct}$. $n$ = 3 independent experiments. Data are shown as mean ± SEM. **e** Gating strategy for flow cytometry analysis of survival and expression of CD25 and FOXP3 in $CD4^{+}CD25^{-}$ and $CD4^{+}CD25^{+}$ enriched populations of T lymphocytes. Debris are first excluded based on FSC-A and SSC-A parameters, then doublets are removed, and live cells (negative for the Live/Dead staining) are further characterized for CD45, CD3 and CD4 expression.

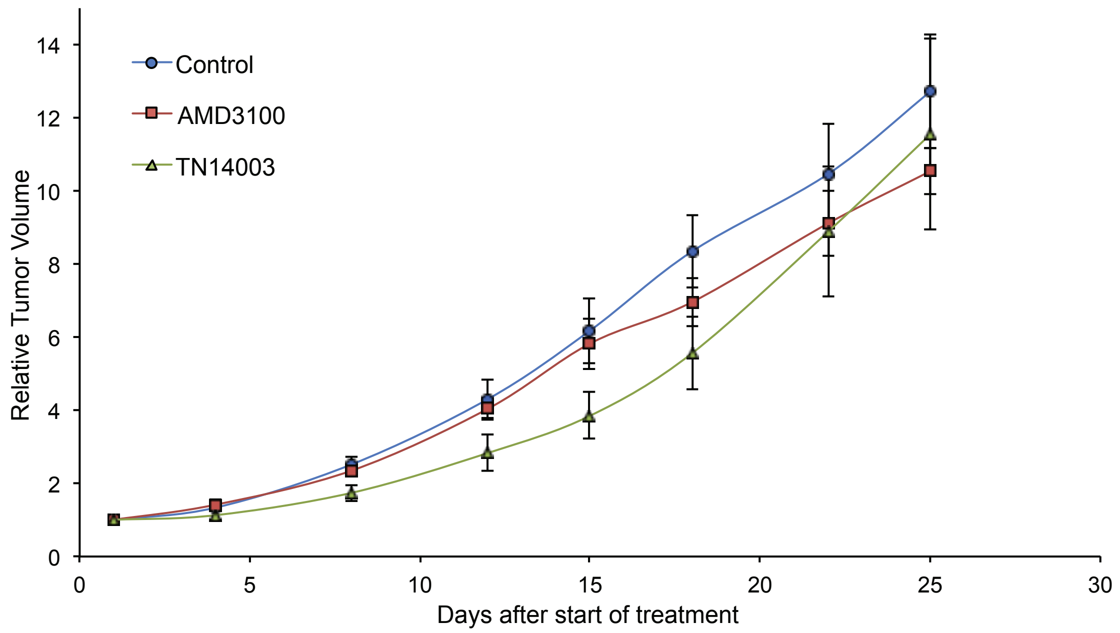


**Supplementary Figure 7** Anti-CXCR4 treatments do not have any impact on tumor growth in a patient-derived xenograft (PDX) model of mesenchymal HGSOC. Relative tumor volume calculated over time, in PDX model of mesenchymal HGSOC. Mice were either untreated (Control, Blue line) or treated 5 days a week by intraperitoneal injection with AMD3100 (7.5mg/kg) (Red line) or TN14003 (7.5mg/kg) (Green line), as indicated. Data are shown as mean ± SEM (*N* = 9 to 10 mice per group). Tumor size was evaluated by measuring two perpendicular diameters of tumors with a caliper, twice a week until they reach ethical size. Individual tumour volumes were calculated as $(V) = a \times b^2/2$, with *a* being the major and *b* the minor diameter. For each tumour, the tumour volume at day n ($V_n$) was reported as the initial volume before inclusion in the experiment ($V_0$) and expressed as relative tumour volume (RTV) according to the following formula: $RTV=V_n/V_0$. Mean and standard error of the mean (s.e.m) of RTV in the same treatment group were calculated, and growth curves were established as a function of time.

| FACS | | | |
|---|---|---|---|
| **Antibody pool for fibroblasts characterization** | Host isotype | Reference | Dilution |
| PerCP/Cy5.5-EPCAM | Mouse IgG2b | Biolegend-324214 | 1/40 |
| APC/Cy7-CD45 | Mouse IgG1 | BD-557833 | 1/20 |
| PE/Cy7-CD31 | Mouse IgG1 | Biolegend-303118 | 1/100 |
| unconjugated-FAP | Mouse IgG1 | R&D Systems-MAB3715 | 1/200 |
| Alexa700-CD29 | Mouse IgG1 | Biolegend-303020 | 1/100 |
| PE-PDGFRβ | Mouse IgG1 | Biolegend-323606 | 1/40 |
| *APC-αSMA* | *Mouse IgG2a* | *R&D Systems-IC1420A* | *1/40* |
| *unconjugated-FSP1* | *Rabbit IgG* | *Abcam-ab27957* | *1/40* |
| *FITC-CAVEOLIN* | *Mouse IgG2b* | *Santa Cruz-sc-70516* | *1/10* |
| **IgG controls** | | Reference | Dilution |
| PerCP/Cy5.5-Mouse IgG2b | | Biolegend-400338 | 1/40 |
| APC/Cy7-Mouse IgG1 | | BD-557873 | 1/20 |
| PE/Cy7-Mouse IgG1 | | Biolegend-400126 | 1/100 |
| unconjugated Mouse IgG1 | | R&D Systems-MAB002 | 1/200 |
| Alexa700-Mouse IgG1 | | Biolegend - 400144 | 1/100 |
| PE-Mouse IgG1 | | Biolegend - 400114 | 1/40 |
| *APC-Mouse IgG2A* | | *R&D Systems-IC003A* | *1/40* |
| *unconjugated Rabbit IgG* | | *Abcam-ab27472* | *1/40* |
| *FITC-Mouse IgG2b* | | *Santa Cruz-sc-2857* | *1/10* |
| **Antibody pool for CD4+ cells characterization** | Host isotype | Reference | Dilution |
| APC-CD4 | Mouse IgG1 | Miltenyi-130-092-374 | 1/20 |
| APC/Cy7-CD45 | Mouse IgG1 | BD-557833 | 1/20 |
| Alexa700-CD3 | Mouse IgG1 | BD-557943 | 1/20 |
| PE-CD25 | Mouse IgG2b | Miltenyi-130-098-211 | 1/20 |
| PE/Cy7-CXCR4 | Mouse IgG2a | Biolegend-306527 | 1/40 |
| FITC-FOXP3 | Rat IgG2a | Ebioscience-11-4776-42 | 1/20 |
| **IgG controls** | | Reference | Dilution |
| PE-Mouse IgG2b | | Miltenyi-130-092-215 | 1/20 |
| PE/Cy7-Mouse IgG2a | | Biolegend-400254 | 1/5 |
| FITC-Rat IgG2a | | Ebioscience-11-4321-41 | 1/100 |
| **Antibody pool for Treg cells sorting** | Host isotype | Reference | Dilution |
| APC-CD4 | Mouse IgG1 | Miltenyi-130-092-374 | 1/20 |
| FITC-CD127 | Mouse IgG1 | Thermofisher-11-1278-42 | 1/20 |
| PE/Cy7-CD25 | Mouse IgG1 | BD-557741 | 1/40 |
| PE-CD45RA | Mouse IgG2b | BD-555489 | 1/20 |

| IHC | | | | |
|---|---|---|---|---|
| Antibody against | Host isotype | Reference | Dilution | Antigen retrieval |
| CXCL12 | Mouse IgG1 | R&D Systems-MAB350 | 1/20 | EDTA pH9 |
| CXCR4 | Rabbit IgG | Epitomics-3108-1 | 1/50 | Citrate pH6 |
| FAP | Rat IgG2a | Vitatex-MABS1001 | 1/100 | Citrate pH6 |
| CD29 | Mouse IgG1 | Abcam-ab3167 | 1/100 | Citrate pH6 |
| PDGFRβ | Rabbit IgG | Abcam-ab32570 | 1/100 | Citrate pH6 |
| αSMA | Mouse IgG1 | Dako-M0851 | 1/400 | Citrate pH6 |
| FSP1 | Rabbit IgG | Abcam-ab27957 | 1/450 | Citrate pH6 |
| CD3 | Mouse IgG1 | Dako-M7254 | 1/90 | Citrate pH6 |
| CD8 | Mouse IgG1 | Dako-M7103 | 1/100 | Citrate pH6 |
| FOXP3 | Mouse IgG1 | Abcam-ab20034 | 1/100 | EDTA pH9 |
| **IgG controls** | | Reference | | |
| Rabbit IgG | | Abcam-ab171870 | | |
| Rat IgG2a | | Affymetrix-14-4321 | | |
| Mouse IgG1 | | Abcam-ab91353 | | |
| Mouse IgG1 | | Dako-X093101 | | |
| Rabbit IgG | | Dako-X090302 | | |

**Supplementary Table 1** List of primary antibodies used in the study. List of primary antibodies used for flow cytometry (upper part), IHC and IF (lower part) analyses. For fibroblast characterization by FACS, antibodies in italic that recognize intra-cellular proteins were not added to the pool in case of analysis of fresh unfixed cells (i.e. for cell sorting). Each antibody was titrated separately to find the best concentration to use. For IHC analyses, appropriate IgG controls were used at the dilution that corresponds to the matched antibody. All antibodies recognize human proteins, and species used for antibody production is indicated.
