# Supplementary material for: miR200-regulated CXCL12β promotes fibroblast heterogeneity and immunosuppression in ovarian cancers
Source: Nat Commun. 2018 Mar 13;9:1056. doi: 10.1038/s41467-018-03348-z (PMC5849633; doi:10.1038/s41467-018-03348-z)
Supplement: Supplementary file 3 — Description of Additional Supplementary Files [file 41467_2018_3348_MOESM3_ESM.pdf]

Description of Additional Supplementary Files

File Name: Supplementary Data 1
Description: CAF-S1 gene signature. List of genes specifically up-regulated (adjusted P value ≤ 0.05) in CAF-S1 fibroblasts isolated from fresh HGSOC samples.

File Name: Supplementary Data 2
Description: CAF-S4 gene signature. List of genes specifically up-regulated (adjusted P value ≤ 0.05) in CAF-S4 fibroblasts isolated from fresh HGSOC samples.
